# Global Research Trend in Vaccine Design

**DOI:** 10.3390/vaccines10122034

**Published:** 2022-11-29

**Authors:** Dharmendra Trivedi, Shanti P. Chaudhari, Atul Bhatt, Manohar Pathak

**Affiliations:** 1 L&T Institute of Project Management, Vadodara 390019, India; 2 School of Liberal Studies, Pandit Deendayal Energy University, Gandhinagar 382007, India; 3 Department of Library & Information Science, Gujarat University, Ahmedabad 380009, India

**Keywords:** vaccine design, bibliometric, scientometric, Scopus, VOS Viewer, R Studio

## Abstract

The current study established a research mapping of the vaccine design using bibliometric indicators and network visualization. For an analysis of the result, the study retrieved a total of 5379 documents from Scopus from 1983 to 2021. The study used the VOS Viewer and the RStudio tools for data visualization. The findings revealed that there has been significant growth in literature on vaccine design in the last two decades; in the last ten years, the year with the most publications were 2020, with 477 publications, and the highest had a total of 14,145 citations. D.R. Burton was ranked as the most prolific author, with 86 publications and 18,449 total citations and was observed as the most frequently published author in the domain. The National Institute of Health (NIH) was the most productive organization in the domain, with 266 publications. The document entitled “Genome analysis of multiple pathogenic isolates of Streptococcus agalactiae” received a total of 1398 citations, and was the most cited document in the field of vaccine design. In network visualization, an analysis of the co-occurrence of keywords showed that “vaccine” and “vaccine design” occurred the most, which was 761 and 335 times, respectively. The study also observed that there were five clusters of author collaboration with a maximum of 18 authors and a minimum of two authors. The findings of the study will aid scholarly coalitions in the domains of medicine and health, information science and bibliometric professionals to carry out further research in the area of vaccine design.

## 1. Introduction

The development of vaccines to prevent infections is no less than a revolution in the history of mankind, and as a result, millions of lives are saved each year from various infectious diseases. Edward Jenner is credited as the inventor of the vaccine, as he developed a vaccine for smallpox in 1796 using a cowpox virus. This was his very first attempt to invent a method to protect against the smallpox disease. The word vaccine is derived from the Latin word vacca, which means cow. However, one study claims that inoculation practices were already being carried out more than 500 years ago by Chinese and Indian traditional medicine practitioners [1]. The development of vaccines is a very sensitive and time-consuming process which is associated with the life and death of the mankind. There are various stages before a vaccine is available for therapeutic use to the public. The earlier vaccine design approach was based on trial and error, which lacked detailed knowledge about the mammalian immune system, but in modern times, advanced information and knowledge are available about the immune system and both general and specific infectious pathogens [2]. The introduction of genetic engineering in vaccine development in the late 20th century greatly impacted the process of vaccine design and as a result, the first vaccine after the advent of genetic engineering was that of hepatitis B and after that, the vaccines for human papilloma virus (HPV), influenza LA and Lyme disease were developed [3]. In 1950, four separate vaccines were available for diphtheria, tetanus, pertussis and smallpox but with the continuous research efforts of scientists, three of these vaccines were included in a single shot. By the mid-1980s, six of the eight vaccines (diphtheria, tetanus, pertussis, measles, mumps, rubella and polio) were integrated into a single shot vaccine for children, and the polio vaccine began to be administered orally. The first and most important stages of vaccine design are basic and applied research, which is conducted by active researchers in the field of vaccine development. It requires rigorous effort to achieve the desired result. Basic research involves the identification and isolation of antigens against specific pathogens, cloning of DNA and the creation of a vector system. Basic research is followed by a clinical evaluation of vaccines, which is completed in various phases. Clinical trials are controlled by a monitoring authority in order to validate the risks and hazards involved in the process. The first phase in the clinical trial of a vaccine is primarily to investigate the safety of the vaccine, determine immunogenicity, the dose and the method of administration of the vaccine to achieve the best immune response, etc. Phase II of a clinical trial is initiated only after the successful completion of phase I. In Phase II, safety and immunogenicity are expanded to check the suitability of the vaccine. Trials of vaccines in Phase II are usually double-blind studies with a placebo-control group. The successful trial of Phase II is followed by a Phase III Clinical trial, which includes thousands of volunteers at risk of acquiring infection. It is a comparatively lengthy process. With the progress that has been made in research and the availability of information, brought about by the application of computational biology (bioinformatics), progress has also been made in vaccinology, resulting in the new domain of immunoinformatics, which aims to develop methods for computational vaccinology, and thus, the development of new vaccines is being accelerated. In silico diagnosis of the intact genome of pathogens, in order to distinguish the gene-determining proteins with elements of useful vaccine objects, is being practiced by the scientists active in vaccine development. This process has been termed reverse vaccinology [4].

### 1.1. Literature Review

There are no previous studies related to the bibliometric mapping of vaccine design research to the best of our knowledge, but there are some studies related to the mapping of vaccine development research related to specific diseases, such as the studies by Guzman et al. [5] of the bibliometric mapping of vaccines in eight Iberian American countries using different indicators such as relationship rate, action index and illustration procedures, group assessment and multi-dimensional grading. Findings showed that Spain and Brazil were in the top two positions in terms of technical development in the area of vaccines, and that public research agencies and universities contributed most in the domain. Garg et al. [6] evaluated malaria vaccine research during 1972–2004 across the world, using various bibliometric indicators. In their study, they reported that the USA, the UK, France and Australia were the most productive countries to publish research on the malaria vaccine. The study suggested that more funding is required for research into a malaria vaccine, and that countries such as India have sufficient research capability and require a more proactive approach for research in this field for their native market. Chen et al. [7] carried out bibliometric studies for a patent of the influenza virus vaccine (IVV) based on technology resources, distribution and development techniques. The findings showed that there is an uneven distribution of technical resources in IVV research, and that the USA has received the largest number of registered patents in the domain, followed by China and Russia. Liu et al. [8] also produced various level assessments of global systematic partnership in influenza virus during 2006–2013. The study analyzed a total of 1878 research papers from the Web of Science. Findings suggested that multi-layered evaluation is useful in global collaboration in the domain. Fernandes et al. [9] carried out the bibliometric analysis of systemic reviews on vaccines and immunizations. The study observed that there was solid growth in the publications on vaccination/immunization during the period from 2008 to 2016. Epidemiology, Safety and Immunology were leading subtopics of research in the domain. Castro et al. [10] investigated global and Latin American scientific literature on pneumococcal vaccines and reported that Brazil, Argentina and Mexico were the most prolific countries that produced the maximum publications in the area of pneumococcal vaccines and a journal entitled “Vaccine” discovered as the most prolific journal in the domain. Zhang et al. [11] reported a bibliometric analysis of the top 100 most cited studies on vaccines. The results highlighted that USA, Switzerland, England and Finland contributed the maximum publications. Out of the top 100 most cited studies, 69 research papers were focused on the prophylactic vaccine. The bibliometric study by Sarirete [12], on the COVID-19 vaccine and sentiment analysis with emphasis on community sentiment, identified that Harvard Medical School and the University of Oxford were the most active institutions in the domain and Medicine and biochemistry subjects produced more papers on the COVID-19 vaccine and the sentiment analysis. Ahmad et al. [13] carried out a similar bibliometric study on the COVID-19 vaccine using a sample size of 916 publications from Web of Science using VOSviewer and Hist Cite as the mapping software. Its findings discovered that the journals entitled Human Vaccine and Immunotherapeutics were the leading journals in the domain followed by Vaccine and Lancet. Among the countries, the USA, India, and the UK produced more research on the COVID-19 vaccine. Ahmad et al. [14] also performed a bibliometric study of the Q fever vaccine based on 478 publications and found that USA and Australia were the most productive countries and Slovak academic of science and INRAE were active institutions in the domain. The most explored research areas include immunology, infectious disease and microbiology in the Q fever vaccine research. Nyeet al. [15] attempted a bibliometric study on NIH-funded HIV vaccine trial network research and reported that PLoS One and the Journal of infectious diseases were highly productive journals in the HVTN-supported research and the most prolific authors are geographically located in the USA followed by South Africa and Thailand. A study undertaken by Mazumdar and Raghul [16] on scientific articles on MMR vaccine design focused on 28 years of Indian contribution on MMR vaccine design. Findings indicated that most of the papers belong to Immunology followed by pediatrics and Medicine research and concluded as a comparative study of MMR vaccine design with global research.

As evident from the above literary output, a significant number of bibliometric studies have been carried out for various types of vaccines but, to the best of our knowledge, no comprehensive bibliometric research is available on vaccine design and thus indicating a vast gap in the quantitative studies of vaccine design.

The current research emphasizes the global research output of overall vaccine design research since its commencement through a bibliometric analysis and visualization. This study reports the research growth in vaccine design from 1983 to 2021 which is a reasonably elongated timespan to see the growth and trend of vaccine design research across the globe.

### 1.2. Major Objectives of the Study

1. To study the distribution of year-wise progress of worldwide research publication, total citation, and average citation pattern in vaccine design research.
2. To find highly productive authors and authors with a creative life in vaccine design research.
3. To know the highly productive organization in the area of vaccine design research.
4. To discover the distribution of highly cited research papers
5. To evaluate network visualization of co-occurrence of author keywords, citation of countries and co-citation of cited authors in the sphere of vaccine design.

### 1.3. Data and Methodology

The bibliometric assessment gives a glimpse of trends and gaps in research in any studied area. The bibliometric studies also deliver the measurement of the quantity of the engraved investigation interfaces in that specific arena of research [17]. Worldwide, Scopus is one of the most prevalent, reliable and prominent citation databases and many researchers have used this database for bibliometric analysis [18,19,20]; hence, the present study used the Scopus database for bibliometric analysis. The data relating to vaccine design has been retrieved from the Scopus database in the 3rd week of May 2022. The following search keywords were applied: TITLE-ABS-KEY (“Vaccine design”) AND (EXCLUDE (PUBYEAR, 2022)). A total of 5379 results were retrieved for the years 1983–2021. These results were downloaded into an MS-Excel file and all the records were checked manually in order to check if any irrelevant records exist. All the author and organization name variants were standardized to common names in all the records in the metadata. The bibliometric network analysis was applied repeatedly for the evaluation of the quality of published work, research developments, global progress of nations, scientific research organization, the network visualization of keywords in five years block were carried out to notice the shift in themes of research in vaccine design. Network visualization of authors s also carried out using the VosViewer software. VOS Viewer© can exhibit a diagram in distinct aspects like intensity view, assemble and sprinkle view [21]. As in contemporary research, many of the researchers used the VOSViewer© tool for visualization and the mapping of research in the various bibliometric studies [22,23,24,25,26]. The bibliometric indicators findings are described in the horizontal format and the total link, clusters and link strength are explained by applying VOSViewer© and presented in an illustrative manner. The present study also used RStudio for the analysis of the author’s productive life and author collaborations in vaccine design research.

## 2. Results

### 2.1. Distribution of Yearwise Progress of Publications, Total Citation and Average Citations

The study evaluated the annual growth of publications, total citations and average citations per publication in the subject domain of vaccine design. Table 1 shows that the publications were started in 1983 and it observed a significant growth after the year 2002. During the years 2002 to 2011, a 10-year span, there were 1570 papers published in the domain and it increased more than double in the last ten year, i.e., 2012 to 2021, with a total of 3263 publications. The study also found that in 2020, a maximum of 14,145 total citations were received in the last ten years of vaccine design publications. In the year 2013, a maximum of 42.58 average citations per publication and in the year 2014, the highest 51 h-index was reported in the last ten years of publications.

### 2.2. Distribution of Most Prolific Authors

**Figure 1:**
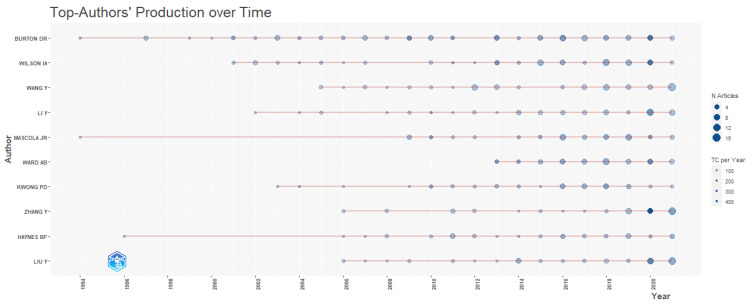
Author Productive life.

Table 2 depicts the most prolific authors in vaccine design research publications. It is observed that D R Burton is the most productive author in vaccine design publication with 86 publications, he also received the maximum number of citations, i.e., 18,449 total citations followed by “IA Wilson”, who contributed 63 publications with 7926 citations and A B Ward with 48 publications and 4814 total citations. The study also showed that all the top ten most prolific authors are geographically located in the USA and two of the then authors are affiliated with MIT (Massachusetts Institute of Technology, Cambridge, MA, USA) and two authors from SRI (Scripps Research Institute, San Diego, CA, USA). The analysis of the author’s productive life using R Studio in vaccine design research as shown in Figure 1 revealed that D R Burton and “IA Wilson” published frequently and received a significant number of citations too in their research publications. 

### 2.3. Distribution of Highly Productive Organizations

The analysis of the most productive organizations in the field of vaccine design has been presented in Table 3, which shows that NIH (National Institute of Health, Bethesda, MD, USA) published a maximum of 266 publications followed by SRI (Scripps Research Institute, La Jolla, CA, USA) with 213 publications and the NIAD (National Institute of Allergy and Infectious Diseases, North Bethesda, MD, USA) with 197 research publications. This Study reports that SRI also received a maximum of 26,434 total citations and a 74 h index; whereas, the University of Melbourne received the lowest of 5066 total citations with 34 h in highly productive organizations. The cited ratio (CR) was calculated based on the total publications cited at least once. Massachusetts Institute of Technology (MIT) received the top position with 99.2 among the highly productive organization in the cited ratio analysis. The USA is the dominant country with eight organizations. There is one organization each from UK and Australia among highly productive organizations.

### 2.4. Distribution of Highly Cited Documents

Analysis of the most highly cited documents has been shown in Table 4. A maximum of 1398 citations were received by the paper entitled “Genome analysis of multiple pathogenic isolates of streptococcus” by Tettelin, H., et al., published in Proceedings of NAS from USA. The publication entitled “Rational design of envelope identifies broadly neutralizing human monoclonal antibodies to HIV-1” by Wu, X., et al. received a maximum of 106.67 average citations per year published in the journal Science. Out of the ten most highly cited document, six documents received 1100+ citations each. Four of the ten most highly cited papers were published in the journal Science and two papers were published in the Proceedings of NAS.

### 2.5. Analysis of Bibliometric Visualisation

#### 2.5.1. Citation Analysis When Unit of Analysis Is Countries

**Figure 2:**
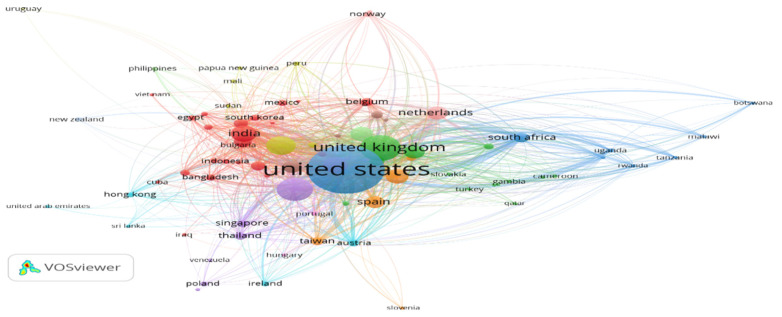
Citation analysis when unit of analysis is Countries.

Figure 2 represents the network visualization of the citation analysis when the element of analysis is a country. The criterion for generating the network was set with countries having at least two citations and a minimum of four documents. There were 81 countries meeting the criteria based on these countries and they were grouped into 13 clusters and 1112 links with total link strength of 20,653. By going through the network map on countries based on the citation, it was observed that the USA received a maximum citation of 127,487 with 12,194 link strength followed by the UK with 37,509 total citations and 4791 total link strength and Australia with 14,260 total citations with 1753 link strength ranking second and third, respectively.

#### 2.5.2. Network Visualisation of Co-Citation of Cited Authors

**Figure 3:**
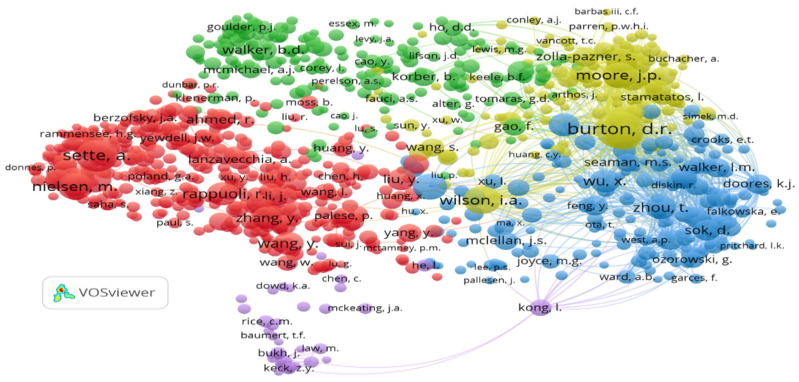
Network visualization of co-citation of cited authors.

In order to find out the most cited authors in vaccine design research during the epoch, we set the criteria with authors having at least the minimum five citations. A total of 1191 of 252,560, authors meet the threshold. Further, it was observed that they have been grouped into five clusters with 409,123 links. The top three cited authors are “Burton, D.R.” (419,967 Link strength), “Kwong, P.D.” (227,336 Link Strength) and “Moore, J.P.” (222,480 Link Strength) (Figure 3).

#### 2.5.3. Network Visualization of Co-Occurrence of Keywords

**Figure 4:**
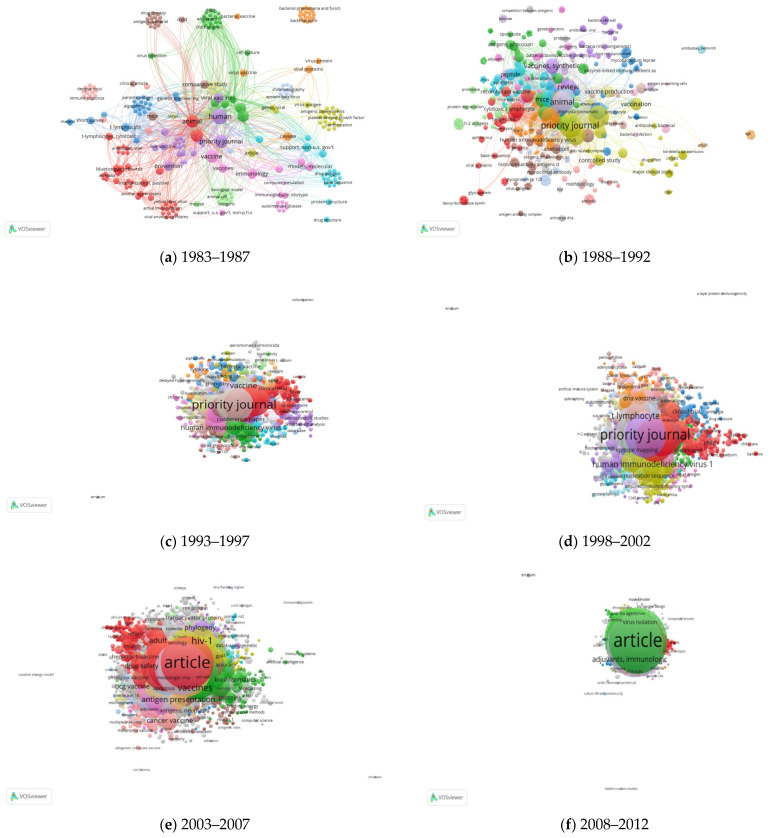

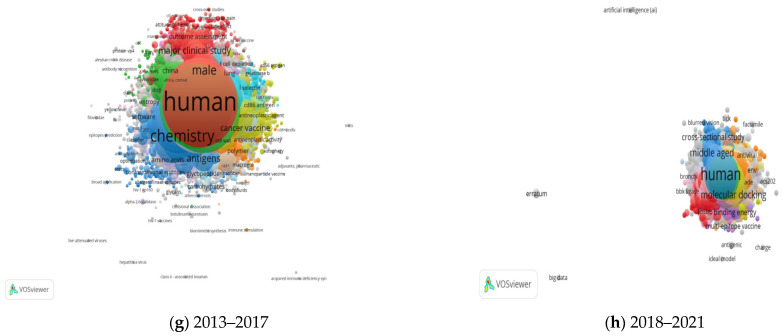
Network visualization of co-occurrence of keywords.

Keywords are an important aspect of knowing the themes of research over a period of time. Based on the author’s keyword network, a map of keywords was drawn (Figure 4a–h) as long as they had a minimum of five occurrences. In the research papers, the author provides a keyword associated with the subject they included in their research work, and it will aid others in the scholarly community. The author’s keyword assessment offers developments on the examination of contemporary times. Figure 4a–h describes the co-occurrence of keywords employing the VOSviewer© tool. Out of the total 7991 author keywords, 522 keywords meet the threshold with a minimum of five occurrences. Two keywords are nearer to each other if they co-occurred in published articles more frequently. An analysis revealed that there were 15 main clusters, 7224 links and total link strength of 11,840 were reported. It is observed that “vaccine” and “Vaccine design” has a maximum occurrence of 761 and 335 times and has the highest link strength of 2061 and 722, respectively. In addition to that “epitope” (156 occurrences), “SARS-CoV-2” (153 occurrences) and “HIV-1” (137 occurrences) were in the top five ranking in co-occurrence of keywords analysis. During 1983–1987, there are a total of 275 keywords grouped into 13 clusters having 4598 links (Figure 4a); in the epoch 1988–1992, there are 727 keywords scattered into 22 clusters with 16,494 links (Figure 4b); in the year 1993–1997, there are 1667 keywords available which have been grouped into 30 clusters with the largest cluster having 157 items (Figure 4c). During 1998–2002, there are 2924 keywords available with 32 clusters of 126,204 links, the largest cluster is 240 items (Figure 4d). During the time span of 2003–2007, there are 5834 items clustered into 45 groups with 366,803 links. (Figure 4e). The largest cluster has 409 items. During 2008–2012, there are 8427 keywords available divided into 55 clusters (Figure 4f). In the epoch 2013–2017, there are 10,292 keywords available scattered into 58 clusters with 736,130 links. The largest cluster consists of 732 items (Figure 4g). In the last three years, i.e., 2018–2021, there are 10,631 total keywords available, these keywords are grouped into 62 clusters with 793,888 links (Figure 4h). During all the epochs the main keywords are: human, priority journal, animal and immunology. This indicates that in vaccine design research, these are the most frequent keywords that have been used by authors. 

#### 2.5.4. Collaboration Network of Authors

**Figure 5:**
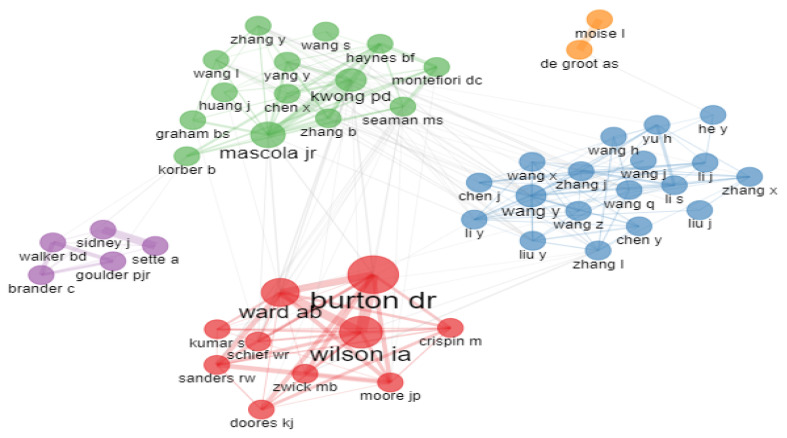
Collaboration Network of Authors.

Collaboration networks, as depicted in Figure 5, show how authors relate to others in vaccine design research. It shows the collaboration network of the leading 49 authors in five different clusters of vaccine design research. It is observed that a prominent collaboration network of a total of 18 authors are in a blue color, followed by 14 author collaborations in a green color, 10 author collaborations network in a red color, five author collaborations in purple color and two author collaborations network in an orange color. The blue, green and red color author collaboration networks also connected with each other in the domain.

## 3. Discussion

The present study evaluates the research productivity of vaccine design using various bibliometric indicators and visualization tools. This study showed that there is an exponential growth in research publications after the year 2002. Almost 61% of the total research publications have come out in the last decade. This study also showed that Burton D.R. and Wilson I.A. secured the top two rankings in the most prolific author category with 86 and 63 publications in vaccine design research. All of the top ten most prolific authors were affiliated with organizations located in the USA and there are two authors affiliated with Scripp’s research institute and Massachusetts Institute of Technology, both in the USA. The top 10 most contributing organizations have cumulatively published 28% of the total research output in vaccine design. Among the organizations, the National Institute of Health (NIH) secured the top position with 266 publications followed by Scripps Research Institute with 213 publications. On the basis of average citation per publication, Howard Hughes Medical Institute is the most prolific organization having 108.08 average citations per publication. Among the list of top ten institutes, eight institutes are geographically located in the USA and one each from the UK and Australia. Among the highly cited publications, it has been observed that three documents received 100+ average citations per year (ACPY). The highly cited document was published in the journal Science (four publications) and the Proceedings of the national academy of sciences of the USA (two publications). On the basis of results explained above, it can be concluded that, during the last 20 years, research in vaccine design has shown significant growth in the number of publications and most of the research is concentrated in the USA. In the analysis of the co-occurrence of author keywords, it is reported that “vaccine”, “vaccine design”, “Epitope”, “SARS-CoV-2” and “HIV-1” secured the top five keywords in the domain. The study reported a total of five cluster collaborations of authors in the vaccine design domain with a maximum of 49 authors clusters and a minimum of two author clusters.

### Research Implications

A bibliometric assessment endeavor to bring a glimpse of the overall research landscape in any particular subject domain over a period of time. The bibliometric study has evidently occurred as a significant technique for assessing systematic production and it has an increasing influence on all the disciplines of research [25]. The current investigation is an attempt to provide a ready reference source for researchers, policy-makers, Librarians, Journal editors and other stakeholders in vaccine design research. The analysis offers an insight into the scholarly community on authors, country and institutional collaboration that can be useful as a policy document for intellectual coalitions. This study is an aid for funding agencies to prioritize the researchers. This study is also helpful for researchers of medicine and health, Bibliometrics, Scientometric and Information Sciences professionals to understand research drifts and learn productivity in terms of invention, influence and association.

## 4. Conclusions and Direction of Future Research

As vaccine design is a collective determination where scientists, governments or scientific research organizations are working day and night to save human lives, we believe that the findings of this study must be helpful for the entire research community for collaboration, and additional research should be done in the domain to bridge the gap in research. However, this research has reported new information in vaccine design research but also has some constraints as the current study has considered the Scopus database, because of which the studies published in the journals not indexed in Scopus will be missing from this analysis. A further study using PubMed, WoS (Web of Science), Google Scholar (GS) and other databases is recommended with a larger dataset for microanalysis of the research landscape in vaccine design. The current study alone cannot be measured as an auxiliary for other evaluation techniques (For Example. meta-analysis or systematic review). Hence, we should deliberate this bibliometric analysis as an initial step towards more inclusive assessment in vaccine design research.

## Figures and Tables

**Table 1:** Distribution of year-wise growth of publications, total citations and average citation.

Year	Total Publications (TP)	Total Citations (TC)	Mean	Average Citation Per Publications (ACPP)	h-Index
1983–1991	84	4407	62.51	52.46	26
1992–2001	462	29,131	60.01	63.05	88
2002–2011	1570	91,249	59.96	58.12	139
2012	235	8100	3.45	34.47	47
2013	237	10,092	4.73	42.58	50
2014	279	9199	4.12	32.97	51
2015	270	8007	4.24	29.66	49
2016	271	8627	5.31	31.83	46
2017	277	6307	4.55	22.77	41
2018	341	7552	5.54	22.15	43
2019	331	5444	5.48	16.45	36
2020	477	14,145	14.83	29.65	45
2021	545	3499	6.42	6.42	27
**Total/Average**	**5379**	**205,759**	**18.55**	**38.25**	

**Table 2:** Distribution of the most prolific authors.

Author Name	TP	TC	h-Index	ACPP	Affiliated Institution	Country
Burton, D.R.	86	18,449	57	214.52	Massachusetts Institute of Technology	USA
Wilson, I.A.	63	7926	37	125.81	Scripps Research Institute	USA
Ward, A.B.	48	4814	28	100.29	Scripps Research Institute	USA
Sette, A.	39	3267	23	83.77	Aligning Science Across Parkinson’s (ASAP)	USA
Mascola, J.R.	38	5264	31	138.53	National Institute of Allergy and Infectious Diseases (NIAID)	USA
De Groot, A.S.	37	952	14	25.73	University of Rhode Island	USA
Kwong, P.D.	37	5277	30	142.62	Columbia University	USA
Sanders, R.W.	34	3582	18	105.35	Weill Cornell Medicine	USA
Walker, B.D.	32	4528	26	141.5	Massachusetts Institute of Technology	USA
Montefiori, D.C.	28	1993	19	71.18	Duke University Medical Centre	USA

**Table 3:** Distribution of Highly Productive Organizations.

Affiliations	TP	TC	h-Index	CR	ACPP	Country
National Institutes of Health NIH	266	17,243	68	96.6	64.82	USA
Scripps Research Institute	213	26,434	74	97.7	124.1	USA
National Institute of Allergy and Infectious Diseases NIAID	197	13,612	58	97	69.1	USA
University of Oxford	163	11,081	53	93.3	67.98	UK
Harvard Medical School	141	13,550	57	97.9	96.1	USA
Massachusetts Institute of Technology	132	12,289	54	99.2	93.1	USA
Massachusetts General Hospital	132	12,640	58	98.5	95.76	USA
University of Washington	110	11,309	46	97.3	102.81	USA
University of Melbourne	95	5066	34	99	53.33	Australia
Howard Hughes Medical Institute	72	7782	44	95.8	108.08	USA

**Table 4:** Distribution of the highly cited documents.

Article Title	TC	Name of Source	Year	ACPY
Genome analysis of multiple pathogenic isolates of Streptococcus agalactiae: Implications for the microbial “pan-genome” by Tettelin, H. et al.	1398	Proceedings of the National Academy of Sciences of the United States of America	2005	82.24
Broad and potent neutralizing antibodies from an African donor reveal a new HIV-1 vaccine target by Burton, D.R. et al.	1350	Science	2009	103.85
Rational design of envelope identifies broadly neutralizing human monoclonal antibodies to HIV-1 by Wu, X. et al.	1280	Science	2010	106.67
Synthetic peptide vaccine design: Synthesis and properties of a high-density multiple antigenic peptide system by Tam, J.P.	1184	Proceedings of the National Academy of Sciences of the United States of America	1988	34.82
Interferons and viruses: An interplay between induction, signaling, antiviral responses and virus countermeasures by Randall, R.E. et al.	1171	Journal of General Virology	2008	83.64
T-cell quality in memory and protection: Implications for vaccine design by Seder, R.A. et al.	1135	Nature Reviews Immunology	2008	81.07
Broad neutralization coverage of HIV by multiple highly potent antibodies by Walker, L.M. et al.	1113	Nature	2011	101.18
Efficient neutralization of primary isolates of HIV-1 by a recombinant human monoclonal antibody by Burton, D.R. et al.	962	Science	1994	33.17
A neutralizing antibody selected from plasma cells that bind to group 1 and group 2 influenza A hemagglutinins by Corti, D. et al.	852	Science	2011	77.45
CD8+ T-cell responses to different HIV proteins have discordant associations with viral load by Kiepiela, P. et al.	824	Nature Medicine	2007	54.93

## Data Availability

Not applicable.

## Abbreviations

NIH	National Institute of Health
HPV	Human Papilloma Virus
DNA	Deoxyribonucleic acid
IVV	Influenza virus vaccine
MMR	Measles, Mumps, Rubella
HVTN	HIV Vaccine Trials Network
INRAE	National Institute for Agriculture, Food and the Environment (France)
TP	Total Publications
TC	Total Citations
ACPP	Average Citation per Publication
SRI	Scripps Research Institute
NIAD	National Institute of Allergy and Infectious Diseases
CR	Cited Ratio
ACPY	Average Citation per year
